# New insight into spatial ecology of Griffon Vulture (*Gypsfulvus*) on the Balkans provides opportunity for focusing conservation actions for a threatened social scavenger

**DOI:** 10.3897/BDJ.9.e71100

**Published:** 2021-08-23

**Authors:** Hristo Peshev, Atanas Grozdanov, Elena Kmetova–Biro, Ivelin Ivanov, Georgi Stoyanov, Rigas Tsiakiris, Simeon Marin, Saša Marinković, Goran Sušić, Emanuel Lisichanets, Irena Hribšek, Zoran Karić, Sven Kapelj, Lachezar Bonchev, Emilian Stoynov

**Affiliations:** 1 Fund for Wild Flora & Fauna, 49 Ivan Mihaylov Str., office 327, P.O.Box 78, www.fwff.org, pirin@fwff.org, Blagoevgrad, Bulgaria Fund for Wild Flora & Fauna, 49 Ivan Mihaylov Str., office 327, P.O.Box 78, www.fwff.org, pirin@fwff.org Blagoevgrad Bulgaria; 2 South-West University „Neofit Rilski“, Faculty of Mathematics and Natural Sciences, Department of Geography, Ecology and Environmental Protection, Blagoevgrad, Bulgaria South-West University „Neofit Rilski“, Faculty of Mathematics and Natural Sciences, Department of Geography, Ecology and Environmental Protection Blagoevgrad Bulgaria; 3 Department of Zoology and Anthropology, Faculty of Biology, Sofia University “St. Kliment Ohridski”, 8 Dragan Tsankov Blvd, zootribe@gmail.com, Sofia, Bulgaria Department of Zoology and Anthropology, Faculty of Biology, Sofia University “St. Kliment Ohridski”, 8 Dragan Tsankov Blvd, zootribe@gmail.com Sofia Bulgaria; 4 Austrian Ornithological Central, Vienna, Austria Austrian Ornithological Central Vienna Austria; 5 Green Balkans – www.greenbalkans.org, 9 Stara Planina Str., Stara Zagora, Bulgaria Green Balkans – www.greenbalkans.org, 9 Stara Planina Str. Stara Zagora Bulgaria; 6 Central European University, Department of Environmental Sciences and Policy, Vienna, Austria Central European University, Department of Environmental Sciences and Policy Vienna Austria; 7 Birds of Prey Protection Society, www.bpps.org, Sofia, Bulgaria Birds of Prey Protection Society, www.bpps.org Sofia Bulgaria; 8 Ministry of Environment and Energy, Forestry Service of Ioannina, Ioaninna, Greece Ministry of Environment and Energy, Forestry Service of Ioannina Ioaninna Greece; 9 Department of Ecology, Institute for Biological Research “Siniša Stanković” – National Institute of Republic of Serbia, University of Belgrade, Bulevar Despota Stefana 142, 11060, Belgrade, Serbia Department of Ecology, Institute for Biological Research “Siniša Stanković” – National Institute of Republic of Serbia, University of Belgrade, Bulevar Despota Stefana 142, 11060 Belgrade Serbia; 10 Ornithological Station Rijeka, Croatian Academy of Sciences and Arts,, Rijeka, Croatia Ornithological Station Rijeka, Croatian Academy of Sciences and Arts, Rijeka Croatia; 11 Nature Conservation Association - AQUILA, Kavadarci, Republic of North Macedonia Nature Conservation Association - AQUILA Kavadarci Republic of North Macedonia; 12 Natural History Museum of Belgrade, Njegoseva 51, Belgrade, Serbia Natural History Museum of Belgrade, Njegoseva 51 Belgrade Serbia; 13 Birds of Prey Protection Foundation, Bulevar despota Stefana 142, Belgrade, Serbia Birds of Prey Protection Foundation, Bulevar despota Stefana 142 Belgrade Serbia; 14 Association BIOM, Zagreb, Croatia Association BIOM Zagreb Croatia

**Keywords:** Griffon Vulture, conservation, GPS tracking, home range, species protection, wildlife movements, Vulture Safe Areas, dynamic Brownian bridge movement model, dBBMM, vulture key zones, Balkan Peninsula

## Abstract

The knowledge in the behaviour and movement of endangered species is of key importance for the precise targeting and assessing the efficiency of nature conservation actions, especially considering vultures, which explore vast areas to locate ephemeral and unpredictable food resources. Therefore, a total of 51 Griffon Vultures (*Gypsfulvus*) from both the re-introduced population and the autochthonous Balkan Peninsula (Balkans) colonies have been tagged with GPS/GSM transmitters in recent years, in order to study their seasonal and spatial distribution. The current study presents the analysis of the high-resolution GPS location data, acquired between January 2016 and March 2021. A total of 1,138,383 locations (an average number of 23,716 ± 18,886 positions per bird, ranged between 2,515 and 76,431 of total fixes per bird; n=48) were used to estimate the home range size and identify the traditional foraging areas and roosting sites of the birds during the wintering, migration/roaming and summering periods. Our results reveal that Griffon Vultures movement activity and home range size varied considerably throughout the annual cycle, especially between their wintering and summering grounds, while exhibiting significant overlapping amongst the tracked individuals. Specifically, immature Griffon Vultures travel long distances across all Balkan Peninsula countries, but always gather with conspecifics, showing strong fidelity to active breeding/roosting sites. The total home range 95% area of the Griffon Vulture population on the Balkans was estimated at 39,986.4 km² and the 50% core area at 1,545.42 km² (n = 48). All tracked birds were found to either visit or frequently use (> 95% of the time) the same seven vulture key zones on the Balkan Peninsula – one in Serbia, one shared between North Macedonia and Bulgaria, one shared between Bulgaria and Greece, two entirely lying in Bulgaria, one in western Greece and one shared between Kvarner Archipelago islands in Croatia and the Julian Alps - Italy, Austria and Slovenia. Several smaller sub-zones were also defined within these general ones. The seven key zones form a coherent network and are used as stepping stones for Griffon Vultures during their migration movements and roaming, but also wintering and summering. The observed concentration tendency of Griffon Vultures on the Balkans and the predictability of their temporal and spatial presence should be used to precisely target, address and substantially increase the efficiency of the conservation measures in this marginal and, thus, still vulnerable meta-population.

## Introduction

Griffon Vulture (*Gypsfulvus* Hablizl, 1783) is an obligate scavenger, gregarious, soaring over large areas for foraging, cliff dwelling bird of prey, formerly widely spread on the Balkan Peninsula (Balkans), but faced a dramatic decline in 20th century (Cramp and Simmons 1980, Demerdzhiev et al. 2007, Andevski 2013, Demerdzhiev et al. 2014, Botha et al. 2017). As a result of wide-scale campaigns for eradication of predators through the use of poisonous baits, in combination with direct persecution and reduction in available food supplies in some areas, around 1980, the population of the species shrunk to ca. 450 pairs in Greece, mainly in Crete (Xirouchakis and Tsiakiris 2009); ca. 200 pairs in former Yugoslavia; and up to only 10 pairs in Bulgaria, becoming locally extinct in Albania and Romania (Cramp and Simmons 1980). In the beginning of the 21st century, despite the successful conservation of the species in Westren Europe - Spain, Portugal and France, where 90% of the European population currently breeds, Griffon Vulture remained marginal, fragmented and threatened on the Balkans with less than 600 breeding pairs (Botha et al. 2017).

Since the 1980s, due to intensification of the conservation activities in Bulgaria, Serbia, Croatia and Crete, the Griffon Vulture increased locally, although some colonies became deserted (e.g. Bosnia and Herzegovina) or continued to decline, remaining on the verge of extinction, primarily in mainland Greece and North Macedonia (Andevski 2013).

Although the places where the species is, or was until recently, breeding on the Balkan Peninsula are generally known by local conservation groups and presented in various technical reports, official documents and local studies (Andevski 2013, Grubac 2014), the spatial distribution and seasonal territory use patterns of Griffon Vultures are still insufficiently known on a regional level, especially in the light of the urgent need for concentration and proper focusing of specific conservation and management efforts.

The movements of Griffon Vultures have been studied elsewhere (Elosegui and Elosegui 1977, Griesinger 1998, Gil et al. 2009) and by other means also on the Balkans (Xirouchakis and Andreou 2009, Susic 2000, Grubac 2014). Those researchers have used ringing and radio transmitters fitted to single birds; metal and colour ring recovery data and wing tagging have all proven that Griffon Vultures and especially the young individuals carry out long-distance migrations. More recently, GPS-based studies on the local territory use and movements of Griffon Vultures from some countries on the Balkans, similar to studies that have been conducted earlier on the Iberian Peninsula and elsewhere (García-Ripollés et al. 2011, Bahat et al. 2001), have also been published for Greece (Crete) (Xirouchakis et al. 2021), Serbia (Hribsek et al. 2021), Eastern Alps and Croatia (Genero et al. 2020) and Bulgaria (Peshev et al. 2018, Stoynov et al. 2018). However, there is still no comprehensive study and detailed analysis of the use of the entire territory, (cross)movements, spatial behaviour and sojourn patterns of the species from the Balkan Peninsula in a regional context. Such a study would possibly reveal the complete picture and provide answers for a list of conservation problems, such as cross-border and local poisoning of different origin (targeting predators, lead- and drug poisoning etc.), electrocution/collision and habitat degradation, all of which have been explicitly highlighted by Botha et al. (2017) as acting on a large scale and thus harder to control.

Griffon Vulture is listed as "Least concern" globally and in Europe in the IUCN Red List (BirdLife International 2017), because of its wide range and a relatively high population number - a total of 648,000-688,000 mature individuals globally, only 10% of which are in Europe. At the same time, the species is regionally listed in all national Red Data Books across the Balkans as "threatened" (Iankov et al. 2015, Susic 2013) and is an object of conservation interest and efforts. Furthermore, the very fast decline of the vulture populations in India between 1990-2000 (Pain et al. 2008), as well as the continuous decline of the entire group in Africa (Botha et al. 2017) justifies the need for preventative actions, timely research and better understanding of the spatio-temporal patterns and any potential conservation implications on related species.

The aim of the current study is to present and analyse the territory use and sojourn patterns of Griffon Vulture, based on high-resolution GPS tracking for the first time on a regional Balkan Peninsula scale. The home range of the species, its core areas, seasonal sojourn and roosting places are revealed on regional and local level and the conservation implications of these findings are discussed.

## Material and methods

In the current study, a total of 51 Griffon Vultures of different ages were equipped with GPS/GSM transmitters in Bulgaria (n = 43), Greece (n = 6) and North Macedonia (n = 2) (Table 1). In order to obtain a more detailed and concise picture, we tagged a total of 20 imported captive individuals, released within local re-introduction projects (Stoynov et al. 2018), a total of 25 wild ones, randomly captured using a hole on the rooftop of existing vulture acclimatisation aviaries (Iezekiel et al. 2003), situated in the areas of Kresna Gorge (UTM FM73), Vrachanski Balkan Nature Park (UTM FN99) and Kotlenska Planina SPA (UTM MH65) in Bulgaria, as well as six individuals, captured in distress in different sites of Greece and North Macedonia and released after rehabilitation.

During the transmitter fitting, the age of the wild-captured birds was determined and recorded by year of hatching using the moulting pattern age determination in Griffon Vulture in line with Zuberogoitia et al. (2013).

The GPS/GSM transmitters (produced by Ornitela UAB - www.ornitela.com) weighed from 30 to 50 g. or < 1% of the body mass of the birds tracked - following the recommendation of < 3% for flying birds (Kenward 2001). The devices were attached either to the birds' lower back by leg-loop harness (OT-30 and OT-50), prepared by three assembled strings (round silicone cord 2 mm + tubular teflon ribbon 0.25" and 0.44") according to Vulture Conservation Foundation (VCF) - internal rules (Daniel Hegglin and Franziska Lorcher - pers. comm.) or pierced to the birds' patagium (OT-P33), together with a vinyl wing-tag. In order to guarantee that the device would fall off in a couple of years, a vulnerable attaching element was deliberately used while fitting. The transmitters were mounted following the best practice in animal welfare - the heads of the birds were covered to ensure minimal stress and the transmitter placement time was reduced to less than ten minutes.

Bird locations were obtained using a global positioning system (GPS), transmitted via a public mobile phone/internet system network (GSM/GPRS). The devices were programmed to save the location data if birds were outside of the coverage area of the given network operator and then to send it once the transmitter was back within range. GPS fixes were acquired every 10 min during the day (between 0500 and 2000 h UTC+2) with dormancy periods during the night. Prior to analysis, the tracking data were inspected and visualised in the Quantum GIS free and open-source cross-platform desktop geographic information system (QGIS.org 2021) to check for outliers and all duplicate coordinates were removed. The data from re-introduced and rehabilitated individuals were used after the 50th day following their release into the wild, to avoid bias due to re-acclimatisation. Only locations taken in the interval between 0600-1800 h UTC+2 within the borders of the Balkan Peninsula were used to determine the home ranges, while the rest of the coordinates in the studied hourly range were used for establishing the roosting sites. The location error was less than 20 m.

Only locations from the Balkan Peninusla and the related areas in the Alps were used in the current study, while location data from the Middle East (movement and sojourn), where some of the tracked birds moved for wintering, were excluded. In addition, birds that were tracked for less than 50 days after release were excluded from further analysis. Data from 48 tracked Griffon Vultures were used for calculations. The information presented and analysed was collected in the period 2016-2021.

The four seasons were defined by the winter and summer solstices and spring and autumn equinox dates. The split aimed at best reflecting the life cycle and foraging and sojourn patterns of the tracked individuals and to distinguish between wintering, summering, spring and autumn migration, seasonal residence and breeding (for the adult birds). If a given vulture had only sent fixings for less than 50% of a particular season (< 45 days), the data of that vulture for that incomplete season were not included in the overall calculations. This was done in order to avoid allocating significance to occasional sites only visited a small number of times yet reflected in the shorter data sample.

### Home range estimations

The home range of each vulture was calculated using the dynamic Brownian bridge movement model (dBBMM) (Kranstauber et al. 2012). Statistics were undertaken using R 4.0.3 (R Core Team 2020), the adehabitatHR (v.0.4.18; Calenge 2006, Calenge 2019) and the move (v.4.0.6; Kranstauber 2020) packages.

A 95% dBBMM home range isopleth contour was defined as the general individual home range and 50% dBBMM home range isopleth contour was defined as the core area. We calculated the home ranges for the entire tracking period for all individual birds, as well as the inidividual home ranges for each tracking season. Distinguishing between home ranges used in various times of the year aimed at avoiding the incorrect attribution of high importance sites, where vultures were present for longer periods or throughout the year, as compared to other important sites, however, visited by the birds only in particular parts of the year.

Differences in home range size, seasonal home range size and core area were assessed using one way ANOVA tests and LCD for post-hoc comparisons.

### Defining vulture zones in the Balkan Peninsula

For the aims of the current study, "vulture key zones" were defined using the connectivity and coherence of the spatio-temporal presence of the tracked vultures as follows:

1. Based on connections of the areas of the calculated home ranges from the obtained GPS data, we defined different Griffon Vulture key zones. The total home range was estimated merging all the annual home ranges. The seasonal home ranges were overlapped to highlight the seasonal areas of importance.
2. Based on the frequency of movement of the vultures amongst different areas. If a tracked vulture has spent more than 5% of the tracked days in movement amongst different parts of a given territory, these areas are considered as a single vulture zone, since the visited sites are clearly connected through regular movement.

### Presence and visits of identified vulture zones by different individuals

We analysed the daily movements of the tracked vultures by dividing their daily tracks into three categories: 1. Days in which the vulture was more than 95% of the time/coordinates in the territory of an already-defined zone; 2. Days in which the tracked vulture is in and out of any of the identified zones and 3. Days in which the tracked vulture has been entirely out of any of the identified zones.

## Results and Discussion

The results, presented below, are based on a total of 1,138,383 GPS coordinate locations (an average number of 23,716 ± 18,886 positions per bird, range 2,515 – 76,431 of total fixes per bird); and an average of 62.99 fixes per day, collected over a total of 18,072 days (average 376.5 ± 276.12 per bird, range 50 – 1,160), acquired from a total of forty eight Griffon Vultures tracked between January 2016 and March 2021.

### Home range estimation

Based on the location data described above, the mean 95% home range area for all vultures (n = 48) was calculated at 1,431.22 ± 1,472.12 km² (range 23.2 – 5,320.03 km²). At the same time, the mean 50% core area for all studied vultures (n = 48), was estimated at 30.04 ± 37.58 km² (range 1.2 – 162.79 km²). The total coverage of all vulture core areas on the Balkan Peninsula, obtained by overlapping all acquired 50% polygons, was estimated at 1,545.42 km² and the 95% home range was similarly calculated at 39,986.4 km², which can be considered as the actual range of the Griffon Vulture in the region.

There was no significant difference in home range sizes between the three groups of vultures (Wild caught, Wild/Rehabilitated and Re-introduced) (F = 0.801, df = 2, p < 0.455). The individual home range calculations for the overall duration of the tracking period are provided in Table 1.

### Seasonal home range estimations

Тhe seasonal home range estimations are provided in Table 2. Furthermore, detailed information on the size of the home range of each individual bird for each season is provided as Suppl. material 1.

**Figure 1.**
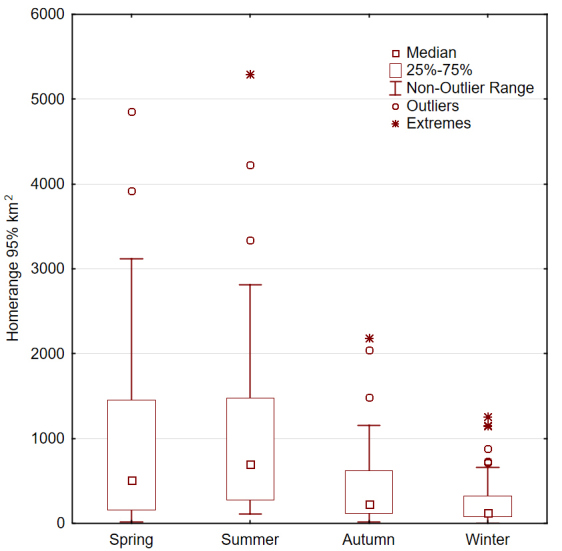
Seasonal home range 95% area in km^2^ of the Griffon Vulture in the Balkan Peninsula.

**Figure 2.**
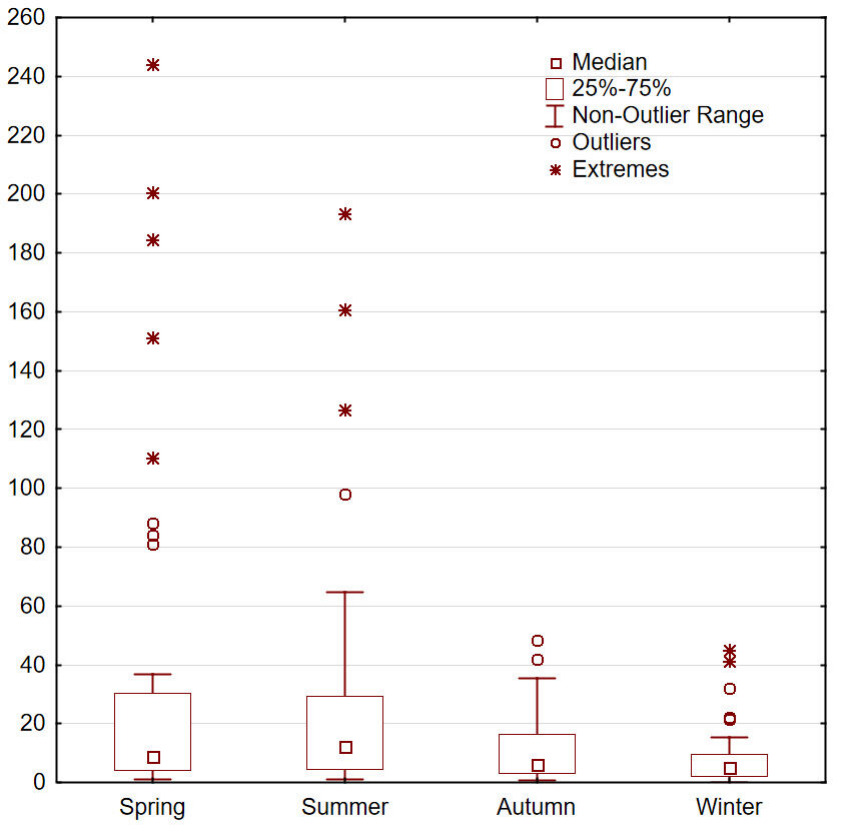
Seasonal 50% core area in km^2^ of the Griffon Vulture in the Balkan Peninsula.

There was a significant difference in the home range sizes amongst the four seasons (F = 11.51, df = 3, p < 0.001; Fig. 1). Post-hoc analysis revealed that home ranges in spring were significantly larger for those in the autumn (p = 0.002) and winter (p = 0.001), but not for those in the summer (p = 0.58). A similar outcome was found for the core areas calculated (F = 5.87, df = 3, p = 0.001; Fig. 2).

### Defining vulture key zones in the Balkan Peninsula

**Figure 3.**
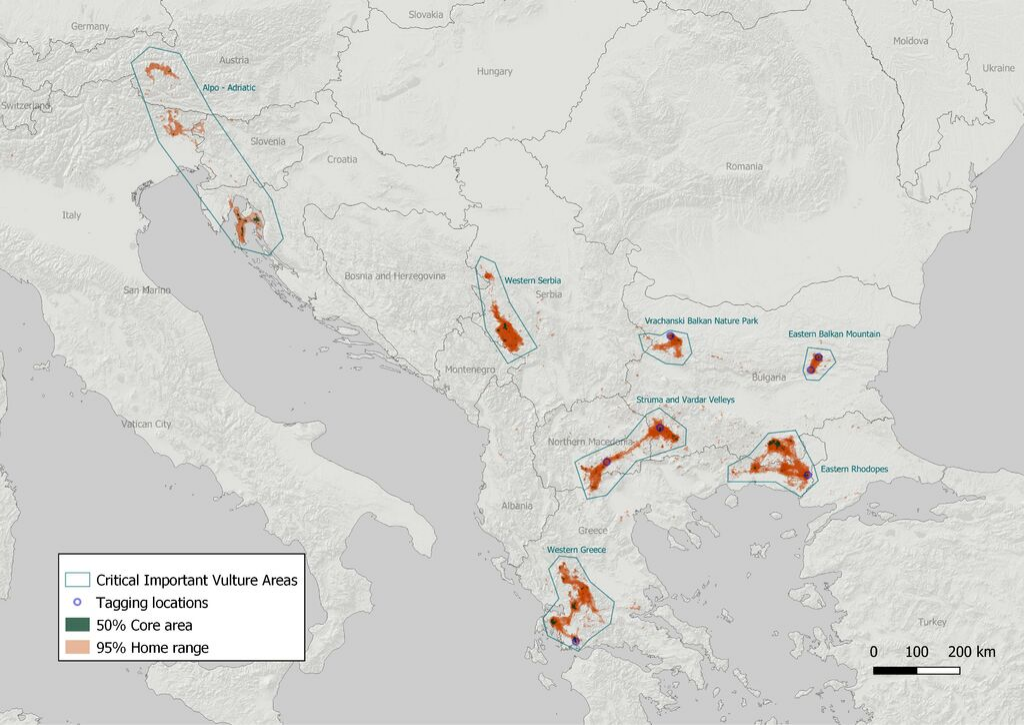
Home ranges 95% and core areas 50%, identifying the Griffon Vulture key zones on the Balkan Peninsula.

Our results reveal that Griffon Vulture movement activity and home range size vary considerably throughout the seasons (see Figs 1, 2), especially between their wintering and summering grounds, while exhibiting considerable overlapping amongst the tracked individuals. Specifically, immature Griffon Vultures travel long distances across all Balkan countries, but always gather with conspecifics, showing strong fidelity to active vulture breeding/roosting sites. In addition to that, all tracked birds prefer to either visit or frequently use (> 95% of the time) the same seven general zones on the mainland Balkans we hereby refer to as "vulture key zones" (hereafter VKZ) – one in Serbia (Western Serbia), one shared between North Macedonia and Bulgaria (Struma - Vardar Valleys), one shared between Bulgaria and Greece (Eastern Rhodopes), two lying entirely in Bulgaria (Vrachanski Balkan Nature Park and Eastern Balkan Mountains), one in Greece (Western Greece) and one shared between Kvarner Archipelago islands in Croatia, the Julian Alps - Italy and Slovenia and Hohe Tauern National Park - Austria (Alpo-Adriatic) (Fig. 3).

Several smaller sub-zones were also defined within the general ones (see Table 3), which might be recognised and managed as actual and potential Vulture Safe Areas (VSA), as proposed in Peshev et al. (2018).

#### 1. Alpo-Adriatic Zone

**Figure 4.**
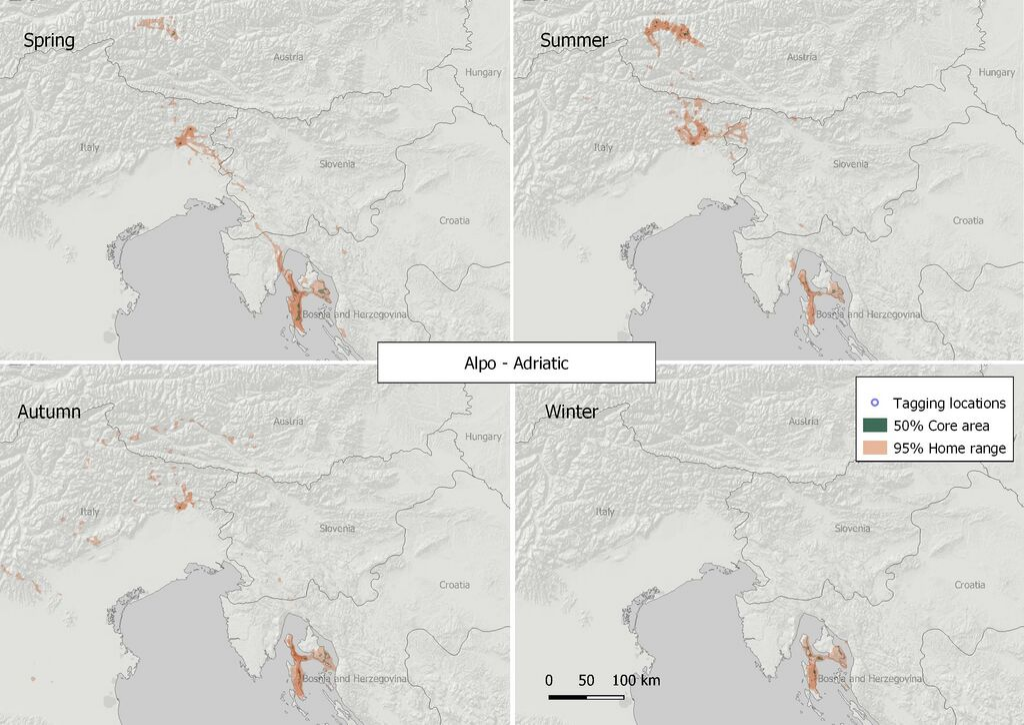
Seasonal home ranges in the Alpo-Adriatic Griffon Vulture key zone.

The VKZ extends beyond the borders of the Balkan Peninusla. It is shared amongst Croatia, Italy, Slovenia and Austria (see Fig. 4) with several centres - Lago di Cornino Nature Reserve (N46.22, E13.02, Italy), where Griffon Vulture has been re-introduced and started breeding in the 1990s (Mihelic and Genero 2005), the Kvarner Archipelago (North Adriatic) - islands of Cres, Krk, Plavnik, Prvich and Pag (N44.98, E14.40, Croatia), Hohe Tauern National Park (N47.14, E12.85, Austria) with some extentions towards the Triglav National Park (N46.36, E13.55, Slovenia) - also reported by Mihelic and Genero (2005) and Genero et al. (2020). The 50% core area of the zone is 291.37 km^2^ and the 95% home range is calculated at 6,803.04 km^2^ (based on the location data of three tracked birds). The sample of tracked birds for this VKZ is small and, although our findings are supported from previously-gathered data (Goran Susic - pers. comm.), but also published studies (Genero et al. 2020), additional research is necessary. A vulture feeding site was operating regularly in Lago di Cornino National Park, Italy and occasionally on the Island of Cres, Croatia, throughout the study period. The active Griffon Vulture breeding colonies within the zone are located in Lago di Cornino in Italy and on the Kvarner Archipelago (North Adriatic) in Croatia (Islands of Cres, Krk, Plavnik, Prvich and Pag).

#### 2. Western Serbia

**Figure 5.**
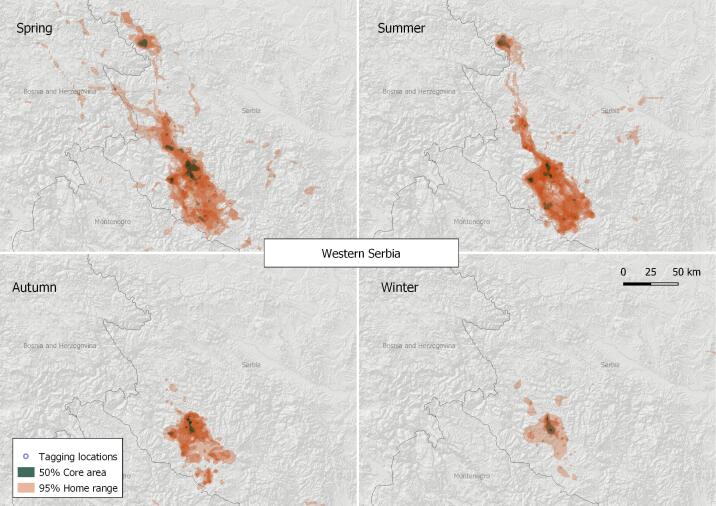
Seasonal home ranges in the Western Serbia Griffon Vulture key zone.

This VKZ lies entirely in Serbia (Fig. 5), centred at the towns of Nova Varos and Sienica along the Uvats River (N43.42, E19.93) and the Treshnitsa River (N44.14, E19.54) where the breeding colonies are. The Griffon Vulture 50% core area, estimated for the zone, is 190.22 km^2^, with a home range of 4,741.83 km^2^ (based on the location data of a total of 10 tracked birds), values above the mean for the Balkan Peninsula. Regular feedings with several tens to hundred of tonnes of slaughter offal/cattle carcasses per year were provided in Uvats Gorge, as well as some less regular feedings were also carried out in Treshnitsa and Mileshevka Gorges (Marinković et al. 2020) during the study period. From the beginning of 2020, another feeding site started operating more to the south - in the area of Pester Plateau Special Nature Reserve, but that one works irregularly.

#### 3. Vrachanski Balkan Nature Park

**Figure 6.**
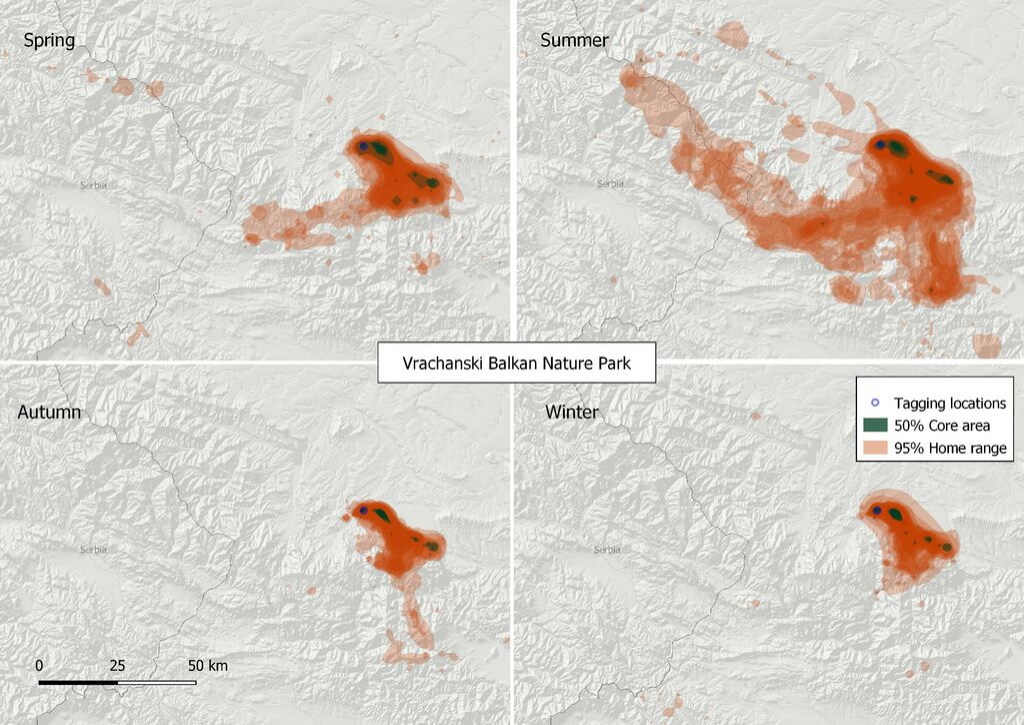
Seasonal home ranges in the Vrachanski Balkan Nature Park Griffon Vulture zone.

This VKZ lies entirely in Bulgaria (Fig. 6), centred near the town of Vratsa (N43.19, E23.52), where active Griffon Vulture breeding colonies were established, following a successful re-introduction project started in 2010. A vulture feeding site, providing some 45-60 tonnes over 200 feeding occasions per year, was operating during the study period near the village of Dolno Ozirovo (N43.25, E23.37). The 50% core area of Griffon Vulture in the zone was estimated at 54.17 km^2^ and the 95% home range is 2,249.32 km^2^, based on the location data of a total of 10 tracked birds.

#### 4. Eastern Balkan Mountains

**Figure 7.**
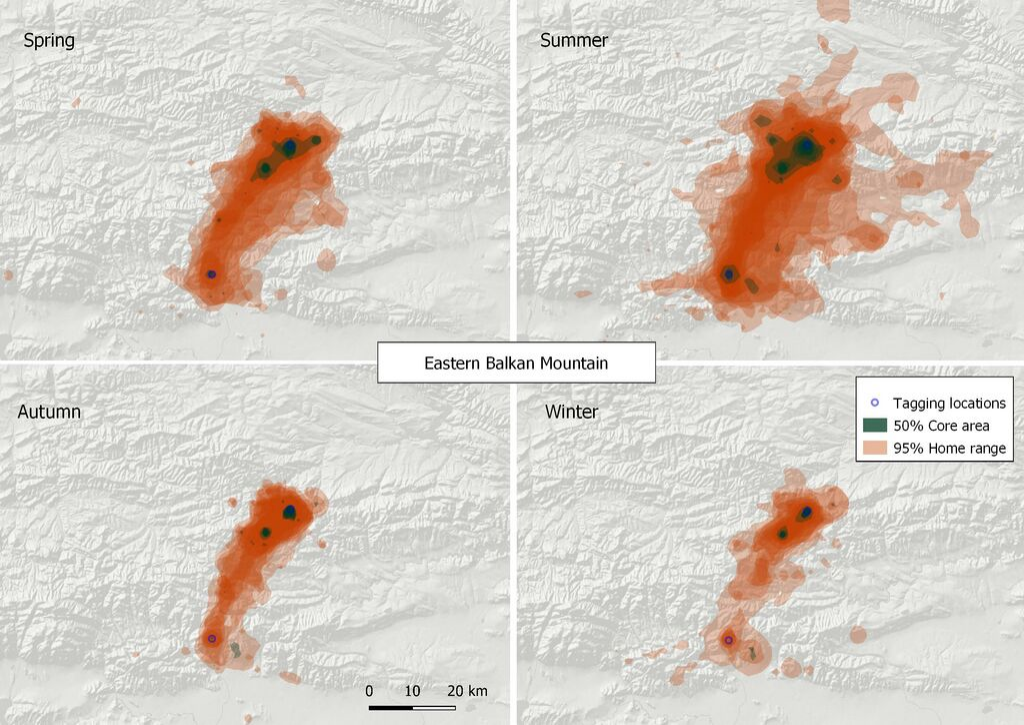
Seasonal home ranges in the Eastern Balkan Mountains Griffon Vulture zone.

This VKZ lies entirely within Bulgaria, centred at the towns of Kotel (N42.88, E26.44) and Sliven (N42.70, E26.34) (Fig. 7), where several small Griffon Vulture breeding colonies were established, following a series of re-introduction projects started in 2010 (Kmetova–Biro et al. 2021). Two vulture feeding sites, each providing some 30-40 tonnes within more than 150 feeding events per year were operating during the study period - one near Kotel (N42.92, E26.46) and another in the Sinite Kamani Nature Park near the town of Sliven (N42.73, E26.30). The Griffon Vulture 50% core area of the zone was calculated at 30.42 km^2^ and the 95% home range at 1,171.38 km^2^ (based on the location data of a total of 22 tracked birds), representing the smallest vulture ranges found on the Balkan Peninsula.

#### 5. Struma and Vardar Valleys

**Figure 8.**
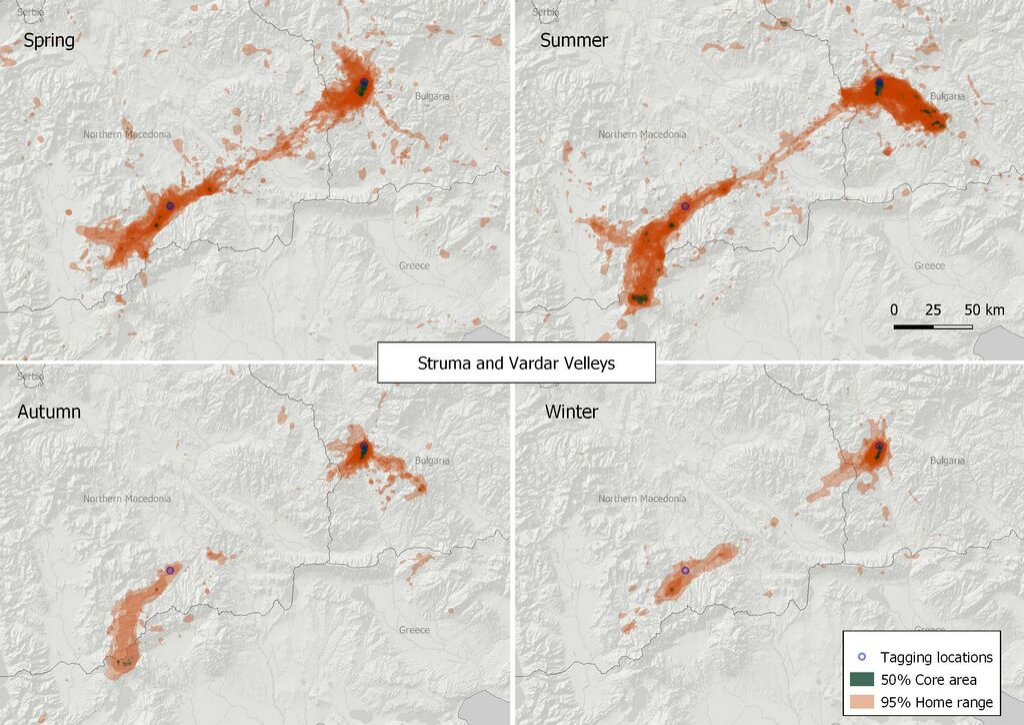
Seasonal home ranges in the Struma and Vardar Valleys Griffon Vulture key zone.

This VKZ is shared between North Macedonia and Bulgaria (Fig. 8), with two centres - Tikvesh area around the town of Kavadartsi - autochthonous colonies (N41.26, E21.96, North Macedonia) and the Kresna Gorge (N41.79, E23.14, Bulgaria), where Griffon Vulture has been successfully re-introduced since 2010 (Peshev et al. 2019). The ranges extend in summer to the high mountain pastures in the area of Kaymakchalan on the border of Greece and North Macedonia (N40.92, E21.78) and Pirin National Park (N41.71, E23.43) in Bulgaria. The 50% core area of the species is calculated at 190.36 km^2^ and the 95% home range is 7,578.93 km^2^ (based on the location data of a total of 31 tracked birds). These are amongst the largest values on the Balkan Peninsula, while at the same time, the zone hosts the smallest number of breeding pairs and constantly-present individuals. Two feeding sites were operating throughout the study period - some 45-60 tonnes of food within more than 200 feeding events per year were provided in Kresna Gorge, Bulgaria (N41.84, E23.16); while some 30-50 feedings with a total of 3-5 tonnes of food per year were occasionally provided in Vitachevo area, North Macedonia (N41.31, E22.50).

#### 6. Eastern Rhodopes

**Figure 9.**
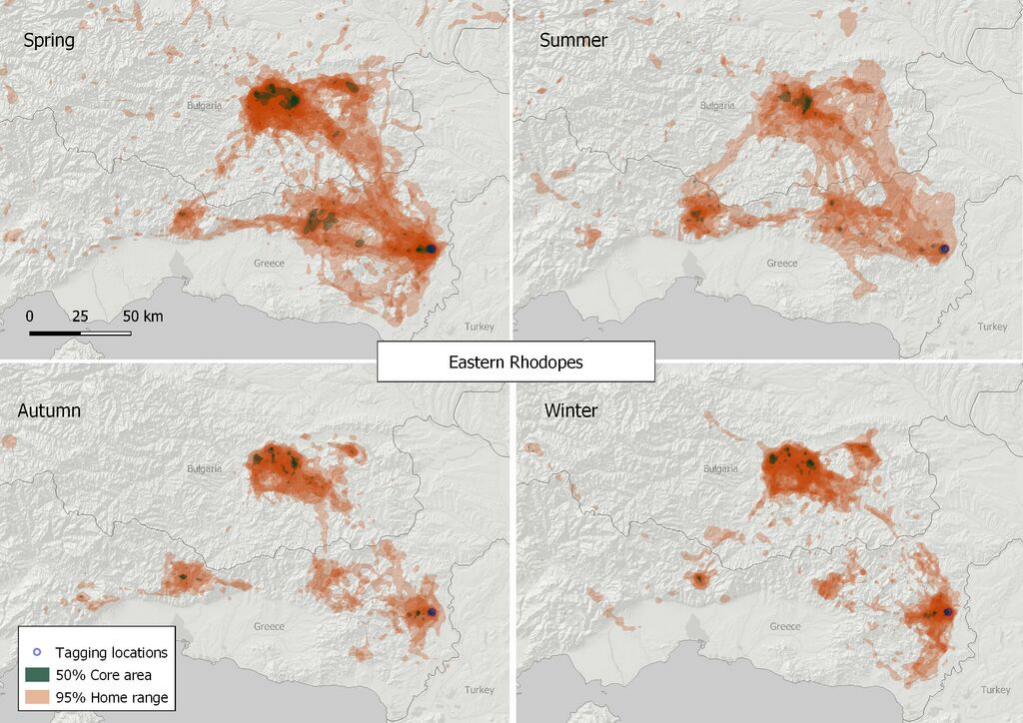
Seasonal home ranges in the Eastern Rhodopes Griffon Vulture zone.

This VKZ is shared between Bulgaria and Greece, centred at the breeding colonies around Studen Kladenets (N41.64, E25.52) and Madjarovo (N41.65, E25.87) in Bulgaria and the Dadia-Lefkimi-Soufli Forest National Park (N41.09, E26.14) and Kompsatos river valley (aka Tracian Metora) (N41.22, E25.15) in Greece. Our location data show that the area around the village of Esochi, Greece (N41.23, E25.77) is more frequently visited for foraging in spring and summer. The 50% core area of the Griffon Vultures in this zone was calculated at 422.63 km^2^ and the 95% home range at 8,371.15 km^2^ (based on the location data of a total of 31 tracked birds), both being the largest estimated on the Balkan Peninsula (Fig. 9). Here, several vulture feeding sites were operating throughout the study period - two in the area of Studen Kladenets (N41.59, E25.64 and N41.62, E25.53), one near Madjarovo (N41.64, E25.87) in Bulgaria and one in Dadia-Lefkimi-Soufli Forest National Park (N41.10, E26.24), each supplied with several tens of tonnes of food per year with a frequency of at least once a week (Arkumarev et al. 2021). Lately, some GPS-tracked Griffon Vultures started visiting the old known breeding site in Nestos Gorge, which was abandoned in 2012 (Andevski 2013), but just recently, a small group with at least one breeding pair recolonised it (Lavrentis Sidiropoulus, pers. comm.) and eventually started to attract migrants and roaming individuals.

#### 7. Western Greece

**Figure 10.**
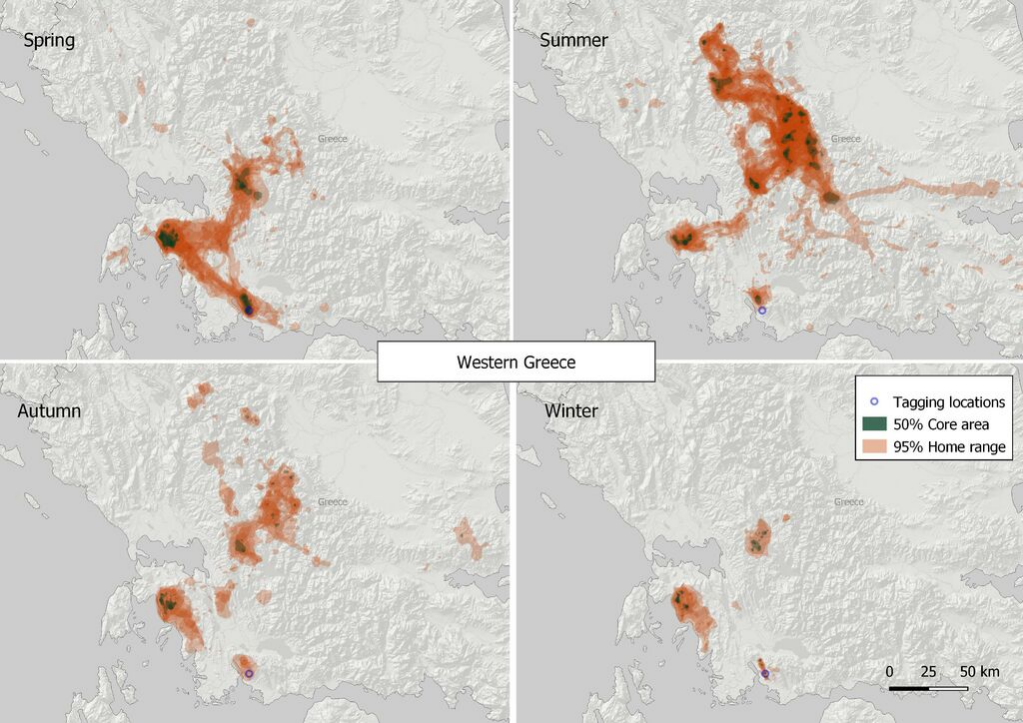
Seasonal home ranges in the Western Greece Griffon Vulture key zone.

This VKZ lies in south-western continental Greece, with three centres - at Messolonghi (N38.50, E21.37), Akarnanika Mountain (N38.74, E20.95) and Embesos (N38.99, E21.34), where local breeding colonies and related wintering sites for vultures from across the Balkan Peninsula are found. The birds were found to move to high mountain pastures of Pindus Mountains with centres in Agrafa (N39.14, E21.69), Tzumerka (N39.44, E21.21) and Karpenisi (N38.94, E21.80) in the summer. The total Griffon Vulture core area 50% of the zone was estimated at 363.54 km^2^ and home range 95% of 7,242.78 km^2^ (based on the location data of a total of 10 tracked birds) being the second largest in the Balkan Peninsula during the study period (Fig. 10). No vulture feeding site was operating in the area during the study period.

### Presence and visits of the respective key vulture zones by different individuals

The studied vultures spent a total of 17,240 days (95.40%) of all days tracked (n = 18,072) entirely in one of the seven key zones identified on the Balkans. In 659 days (3.64% of the time), they were partly in and out of any zone and only in 173 days (0.96%) they were completely outside all the zones outlined.

On the Balkans, Griffon Vultures are less mobile and inhabit smaller home ranges in winter and autumn, as compared to summer and spring, likely related to the fewer daylight hours and the fewer days with suitable weather conditions for soaring flights (Poessel et al. 2017). This explains the higher concentration of Griffon Vultures in winter and autumn that stay in places with active breeding colonies (based on social attraction) and easily accessible well-managed vulture feeding sites (also supported by the study of Arkumarev et al. 2021) if they exist, such as in Lago di Cornino Nature Park in Italy, Uvats Gorge in Serbia, Vrachanski Balkan Nature Park, Kotlenska Planina SPA, Sinite Kamani Nature Park, Kresna Gorge in Bulgaria and the Eastern Rhodopes between Bulgaria and Greece, also in Thrace in the eastern corner of the last country.

The contemporary Griffon Vulture wintering areas in Western Greece are located mainly near wintering free-ranging transhumant livestock herds along the shoreline - Messolonghi, Akarnanika, Embesos and Varasova, where the weather conditions are mild and allow daily flight activities. In this VKZ, even without vulture feeding sites operation, the vultures are concentrated in winter near existing breeding colonies after being eventually halted by a geographic barrier (Mediterranean Sea surrounding the "Greek" Peninsula) during their southward migration in autumn.

In the period spring to early summer (April-June), the vulture movements and flight distances increase, likely due to the improved weather conditions and the more daylight hours. Adult vultures start breeding and this fixes them largely in the colonies as the central place for foraging (Monsarrat et al. 2013), yet many young birds are also concentrated in the same areas, attracted by the availability and easy access to food and the constant presence of the breeding birds. It is known that the vultures use social information about foraging (Cortés-Avizanda et al. 2014) and congregate arround existing colonies of conspecifics.

In the period summer to early autumn (July-October), vultures move to higher parts of the mountains if food is available, concentrating in the following sites: 1. from the Island of Cres and the fore-mountians of the Alps, as well as other parts of the Balkans to Hohe Tauren National Park in Austria and the Julian Alps between Italy and Slovenia; 2. Vrachanski Balkan Nature Park - from fore-mountains to open upland pastures; 3. from Kresna Gorge to Pirin National Park; 4. from Mariovo and Tikvesh to North Macedonia, but also from Kresna Gorge to Kaymakchalan on the border with Greece; and 5. from Messolonghi/Akarnanika/Embesos area to Pindus Mountains (Agrafa, Tzumerka, Karpenisi and others) in Greece. During these months, birds rely less on the vulture feeding sites (supported also by the study of Arkumarev et al. (2021) for the Eastern Rhodopes), because of the ability to fly long distances in search of food and the larger number of accessible carcasses from cattle and sheep (frequently free-ranging) which have been moved out for summer grazing in the mountain pasturelands.

Although vultures travel large distances and cross state borders and various protected areas (Lambertucci et al. 2014), the current study shows that the tracked vultures are mostly spatially and temporally concentrated in seven distinct vulture key zones on the Balkan Peninsula. More importantly, more than 95% of their time, the tracked Griffon Vultures are found in these seven zones year-round and, in case they leave any of them, they return shortly after (two to few days) or move and stay in any of the other key zones identified on the Balkans, unless they move to the Middle East for wintering.

Seasonal home ranges show differences in size and location according to the specific features of the respective zone. Monsarrat et al. (2013) suggest that Griffon Vultures do not forage completely at random, but favour some specific areas. In our study, this is very well visible in the areas with regularly operating permanent vulture feeding sites, which become the centre of activity of the vultures - all fall within the 50% core area of the respective zone. This proves that the national and the pan-Balkan network of vulture feeding sites plays an important role for the Balkan autochthonous and locally re-introduced Griffon Vulture population. Furthermore, this greatly supports our concept for the need of establishment of a network of Vulture Safe Areas (VSA) - similar to Vulture Safe Zones (VSZ) described for South Asia - specifically free of diclofenac (BirdLife International 2014, Botha et al. 2017), but smaller in sizes (in the case of Balkans ~ 50 km^2^), where the full spectrum of threats for the species to be addressed and mitigated on a relatively small territory. This will provide for well-focused management and thus prevent the further reduction of the already vulnerable regional vulture population, especially as most nuclei are located within existing protected areas. Through the maintenance of permanent feeding sites, vultures might be concentrated in such areas and kept safe, while avoiding and managing the threats, such as poisoning, electrocution and collision with overhead cables/wind turbines, which are: 1. acting on vast territories; 2. unpredictable in space and time; and 3. hardly controlled short–term. Such actions are especially important for increasing and safeguarding the survival of the juvenile and immature vultures. These vultures will then move amongst and remain within the VSAs, using them as safe stepping-stones during their migration, roaming, sojourn and roosting across the Balkans.

Out of the known vulture feeding sites in Serbia, Bulgaria, North Macedonia and Greece, the vultures rarely stayed more than an overnight on-passage and were never feeding in other sites, with the exception of several places in Pindus Mountain range that should be given priority for application of urgent vulture conservation measures and to secure them as VSAs, instead of playing roles of ecological traps and thus population sinks (as according to Pulliam 1988). Other seasonally-important places that should be regularly monitored and managed as VSAs, especially during the summer period are: the Kaymakchalan peak on the border between Greece and North Macedonia; the Pirin National Park in Bulgaria; the Valley of Krumovitsa River and the hills to the west of it in the Eastern Rhodopes in Bulgaria; the area between the Dadia-Lefkimi-Soufli Forest National Park and Kompsatos River valley in Greece, with centre around the village of Esochi, the Zlatibor and Pester Plateau in Serbia, the Julian Alps in Slovenia and Italy and the Hohe Tauern National Park in Austria.

Based on the current study and knowledge for the Griffon Vulture's movements and sojourn in Balkan Peninsula, another strategically placed historic breeding/roosting sites should be assessed for their potential to be recolonised by the species (either naturally or assisted) and to be managed in a way as to further enlarge the current network of vulture key zones/safe areas.

## Conclusions

The present research reveals seven well-distinguished key zones for the remnant and locally-re-introduced nuclei of the Griffon Vulture population on the Balkan Peninsula, clearly outlined by the analysis of the collected telemetry data. The monitored individuals spent virtually the whole period of the research (> 95%) in one or more of those seven key zones or in targeted movements between them, demonstrating an extremly high preference for those environmentally suitable areas, involving also species with strong social interactions.

All of the seven identified Griffon Vulture zones on the Balkans offer similar key benefits for the vultures - suitable breeding/roosting sites with gorges, ravines and cliffs nearby; extensive summer and winter pasturelands; presence of conspecifics and all, but Western Greece, have actively managed vulture feeding stations. The seven key zones form a coherent network and are used as stepping-stones for Griffon Vultures during their migration movements and roaming, but also wintering and summering.

The obtained results are of crucial importance for the conservation of the species on the Balkans as they show that the wide range of management efforts could be specifically targeting particular core areas. The knowledge that social scavengers, such as Griffon Vulture, could be spatially concentrated and could be used elsewhere to precisely target adequate management efforts in space and time.

The telemetry-based conclusions are directly related to the concept of VSA and support the urgent need to actively monitor, control and mitigate all risk factors (such as poisoning, electrocution, collision, poaching etc.) and prevent habitat deterioration (e.g. large scale wind farm development, pastureland abandonment) in these well-defined key zones. This is likely the most cost-effective strategy for the conservation of all vulture species during the human-dominated present (the so-called Anthropocene) on the Balkan Peninsula and elsewhere.

The knowledge of the key characteristics of the present Griffon Vulture key zones, identified in Southeast Europe, will assist the potential establishment of new ones, where colonies of the species have existed in the near past. This could help to further increase the coherence of the network of vulture key zones and facilitate the natural dispersal of the metapopulation, lowering the conservation risks in all remaining single sites.

## Supplementary Material

5D4CFFA7-749E-5995-B856-2B16808982A0 10.3897/BDJ.9.e71100.suppl1 Supplementary material 1 Seasonal home-ranges area for all individual birds Data type Area in km2 File: oo_558555.csv https://binary.pensoft.net/file/558555 Hristo Peshev

## Figures and Tables

**Table 1.** Griffon Vulture individuals tracked; GPS transmitter type used; manner of device deployment; age; tagging location; tracking period and number of GPS fixes received per individial. Different groups, based on the type of bird according to the method of capturing and tagging with GPS transmitter, are provided as: 1. "Wild caught" - the bird was a healthy wild individual, native to Balkans, intentionally captured and marked; 2. "Wild/Rehabilitated" - the bird was a wild individual, native to Balkans, captured in distress and rehabilitated and then marked upon release back into the wild; 3. "Re-introduced" - the bird originated from a re-introduction programme - either captive bred in a zoo or translocated after rehabilitation and marked upon release into the wild.

Tag	Transmitter type and model	Tagging location	Year of fledging	Way of capturing to deploy the transmitter	Start date	End date	Received GPS coordinates on the Balkans	Tracking days with data used in calculations	Core area 50%, km^2^	Homerange 95%, km^2^
5 - Petitsata	OT - 30	Kresna Gorge	2012	Wild caught	19.3.2020	26.3.2021	20492	373	8.532	359.259
1H - Wild 1H	OT - P33	Kresna Gorge	2016	Wild caught	12.7.2017	13.5.2018	19996	306	162.79	4176.175
1X - Terziev	OT - P33	Dadia	2017	Wild/ Rehabilitated	22.1.2018	26.3.2021	74306	1160	33.33	2059.875
2H - Wild 2H	OT - P33	Kresna Gorge	2016	Wild caught	12.7.2017	20.6.2020	70453	1075	75.564	2890.259
56 - Survivor	OT - P33	Kresna Gorge	2014	Re-introduced	12.7.2017	20.9.2018	32150	436	33.174	5320.03
A4 - Vrachan	OT - P33	Vrachanski Balkan	2011	Re-introduced	7.11.2017	16.9.2019	32954	678	4.186	320.095
Alexis	OT - P33	Messolonghi	2017	Wild/ Rehabilitated	5.8.2017	27.5.2019	7555	93	91.675	3163.986
B65 - Parvolet	OT - P33	Sinite Kamani	2016	Re-introduced	21.7.2019	27.3.2021	37756	616	10.426	493.88
B69 - Balkan	OT - P33	Kresna Gorge	2016	Wild caught	18.8.2017	6.2.2018	8794	173	24.20	1978.353
B70-UG - Barcelona	OT - P33	Kresna Gorge	2015	Re-introduced	18.7.2017	7.9.2017	4602	52	2.481	395.826
B71-XU - Barca	OT - P33	Kresna Gorge	2015	Re-introduced	3.8.2017	14.10.2017	5429	73	1.437	184.891
BY1- Gorlits	OT - 30	Kresna Gorge	2018	Wild caught	22.2.2020	26.3.2021	24162	399	11.246	2380.805
BY3 - Svetislav 2	OT - 50	Kresna Gorge	2018	Wild caught	22.2.2020	23.1.2021	16874	337	12.737	1434.072
BY7 - Michev	OT - 30	Kresna Gorge	adult	Wild caught	3.6.2020	14.7.2020	3664	50	34.049	909.422
C1-M - Kresna	OT - P33	Vrachanski Balkan	2016	Re-introduced	8.2.2018	16.9.2019	41357	586	4.392	275.712
C5 - Sunchitsa	OT - 50	Kavadarci	2019	Wild caught	30.10.2019	27.3.2021	22230	329	13.736	1951.845
C7 - Svetislav	OT - 50 / OT - P33	Kavadarci/ Kresna Gorge	2019	Wild caught	4.9.2020	26.11.2020	5547	84	31.373	921.962
C9 - Rakitna	OT - P33	Kresna Gorge	2018	Wild caught	24.6.2020	27.3.2021	19015	277	61.214	1823.193
Defile	OT - 30	Kresna Gorge	2016	Wild caught	29.7.2020	26.3.2021	11187	241	5.372	370.085
E1 - Poison detective	OT - P33	Vrachanski Balkan	2014	Wild caught	16.12.2020	27.3.2021	3177	101	2.413	83.473
EX - Extreme	OT - 50	Kresna Gorge	2019	Wild caught	1.2.2021	27.3.2021	2515	55	2.114	23.197
F4 - Stresher	OT - 30	Vrachanski Balkan	2018	Re-introduced	6.12.2019	26.3.2021	22863	477	2.852	143.784
F6 - Zelen	OT - 30	Vrachanski Balkan	2018	Re-introduced	6.12.2019	26.3.2021	27130	477	4.298	191.242
GD-22 -Giannis	OT - 50	Messolonghi		Wild/ Rehabilitated	5.3.2020	27.3.2021	28774	388	72.197	1579.987
GS - Athanasios	OT - 30	Messolonghi	2020	Wild/ Rehabilitated	24.1.2021	27.3.2021	2929	63	9.368	137.276
H1 - Juanjo	OT - P33	Kotel	2016	Wild caught	8.3.2017	28.10.2019	68012	965	1.203	216.167
HW - Struma	OT - P33	Kresna Gorge	2015	Re-introduced	24.6.2020	27.3.2021	18020	277	6.439	320.247
K2M - Mulhouse	OT - P33	Kresna Gorge	2016	Re-introduced	3.8.2017	1.2.2018	11809	183	3.639	570.129
K3A-B2	OT - 30	Messolonghi	2018	Wild/ Rehabilitated	6.3.2020	30.10.2020	14463	239	24.37	470.309
K5M - Baumgart	OT - P33	Kotel	2011	Re-introduced	24.1.2017	27.1.2018	21918	369	3.640	141.962
K7A-B5 - Dinos	OT - 30	Messolonghi	adult	Wild/ Rehabilitated	6.3.2020	26.3.2021	25435	386	106.767	3059.814
K9U - Kotel	OT - P33	Kresna Gorge	2014	Re-introduced	3.8.2017	13.5.2018	19775	284	156.375	5308.372
M2 - Lars	OT - P33	Sinite Kamani	2017	Re-introduced	21.3.2019	19.8.2020	40793	518	6.903	248.354
OX - De Doue	OT - P33	Kresna Gorge	2016	Re-introduced	15.12.2016	18.2.2018	24310	431	37.522	1642.845
P-B2F - Niki	OT - P33	Vrachanski Balkan	2017	Wild caught	10.10.2017	19.2.2018	6951	133	27.995	479.693
V3 - Poison Spy	OT - P33	Kresna Gorge	2017	Re-introduced	13.3.2018	26.3.2021	76431	1097	31.91	4690.052
V5 - Boev	OT - P33	Kresna Gorge	2017	Re-introduced	12.2.2019	24.4.2019	4736	72	1.765	69.608
V8 - Sainte Croix	OT - P33	Kresna Gorge	2017	Re-introduced	3.5.2018	17.12.2019	36784	595	7.128	1290.039
XE - Hemus	OT - 30	Vrachanski Balkan	2017	Re-introduced	1.7.2020	27.3.2021	15157	270	2.853	186.603
XJ - Nikola	OT - P33	Kotel	2015	Re-introduced	15.3.2019	27.3.2021	53929	733	2.851	114.437
Y1 - Gorlitz	OT - P33	Kresna Gorge	2017	Wild caught	24.1.2020	26.3.2021	28916	428	33.858	3042.697
Y2- Whitley (WFN)	OT - 30	Kresna Gorge	2017	Wild caught	22.2.2020	24.3.2021	15968	371	13.978	1481.232
Y4 - Vrachan 2	OT - P33	Kresna Gorge	2018	Wild caught	24.1.2020	6.10.2020	19530	257	34.959	2568.986
Y5 - Alexis 2	OT - P33	Kresna Gorge	2018	Wild caught	1.11.2019	26.3.2021	24041	347	39.265	3889.373
Y6 - Juanjo 2	OT - P33	Kresna Gorge	2018	Wild caught	1.11.2019	26.3.2021	31414	512	41.639	966.519
Y8 - Paris 2	OT - P33	Kresna Gorge	2018	Wild caught	22.2.2020	25.3.2021	19709	393	50.644	2273.77
Y9 - Sinanitsa	OT - P33	Kresna Gorge	2019	Wild caught	24.6.2020	27.3.2021	9061	111	86.372	1907.081
Z7 - Izvor	OT - 30	Vrachanski Balkan	2017	Re-introduced	1.9.2020	27.3.2021	5310	202	4.571	191.373
C2 - Nelson	OT - P33	Kresna Gorge	2018	Wild caught	19.09.2018	26.10.2018	1016	15		
W0818 -Bistritsa	OT - 30	Kresna Gorge	2019	Wild caught	4.9.2020	26.3.2021	2373	33		
A4- Ezerets	OT - P33	Kresna Gorge	2019	Wild caught	4.9.2020	27.3.2021	3536	38		

**Table 2.** Seasonal home range estimations.

Season	Total number of seasons studied for all birds, n	Core area 50%, km^2^					Home range 95%, km^2^				
		mean	median	st. deviation	min	max	mean	median	st. deviation	min	max
Spring	44	34.8602	8.9035	57.947	1.04	244.085	984.604	508.227	1120.12	14.837	4855.93
Summer	55	26.2727	12.21	37.9947	1.233	193.352	1033.56	696.312	1041.45	110.111	5292.27
Autumn	50	11.5356	6.305	11.7446	0.867	48.415	421.864	225.127	477.72	16.452	2187.2
Winter	56	7.76018	5.1155	9.12612	0.096	44.963	258.682	129.543	298.938	2.1	1256.35

**Table 3.** Griffon Vulture zones on the Balkans, core areas and home range sizes, sub-zones, type of presence and food sources utilised in the given area (based on field observations and available expert data).

Nº	Vulture key zone/ Country	Vultures located in the zone	Area used by vutures, 50% core area, km^2^	Area used by vutures, 95% Home range, km^2^	Sub-zone(s) within the main site	Type of presence	Food resources used by vultures - Feeding sites (FS) place/name
1	Alpo - Adriatic, Austria/ Italy/ Croatia	K7A-B5, V3, Y8, (n=3)	291.37	6803,04	Kvarner Archipelago, Croatia (islands of Cres, Krk, Plavnik, Prvich and Pag)	Breeding	Year-round free grazing livestock
					Lago di Cornino Nature Park, Italy	Breeding and summering	Feeding site
					Hohe Tauern National Park, Austria	Summering	Summer livestock grazing
2	Western Serbia, Serbia	56, B69, BY3, K9U, V3, Y1, Y2, Y6, Y8, Y9, (n=10)	190.22	4741.83	Uvats Gorge, Mileshevka Gorge, Treshnitsa Gorge	Breeding, wintering, summering, on passage	Uvats Gorge FS, Treshnitsa Gorge FS, Year-round grazing livestock
3	Vrachanski Balkan Nature Park, Bulgaria	1X, 56, A4, C1-M, C5, F4, F6, P-B2F, XE, Y1, Z7, E1 (n=12)	54.17	2249.32	Vrachanski Balkan	Breeding, wintering, summering, on passage	Vrachanski Balkan FS, Year-round grazing livestock
4	Eastern Balkan Mountain, Bulgaria	1X, B65, H1, K5M, M2, V3, XJ, Y1, Y5, (n=9)	30.416	1171.38	Kotlenska Planina SPA	Breeding, wintering, summering, on passage	Kotel FS, Year-round grazing livestock
					Sinite Kamani Nature Park	Breeding, wintering, summering, on passage	Sinite Kamani FS, Year-round grazing livestock
5	Struma and Vardar Velleys, Bulgaria/ North Macedonia	1H, 2H, 5, 56, A42020, B69, B70-UG, B71-XU, BY1, BY3, BY7, C5, C7, C9, DEFILE, EX, HW, K2M, K9U, OX, V3, V5, V8, W0818, Y1, Y2, Y4, Y5, Y6, Y8, Y9, (n=31)	190.36	7578.93	Kresna Gorge	Breeding, wintering, summering, on passage	Kresna Gorge FS, Year-round grazing livestock
					Pirin National Park	Summering	Summer livestock grazing
					Demir Kapiya, Tikvesh and Mariovo	Breeding, wintering, summering, on passage	Vitachevo FS, Year-round grazing livestock
					Kaymakchalan	Summering	Summer livestock grazing
6	Eastern Rhodopes, Bulgaria/ Greece	1H,1X, 2H,56, A42020, Alexis, B69, BY1, BY7, C5, C7, C9, H1, K9U, P-B2F, V3, V8, W0818, Y1, Y4, Y5, Y9, (n=22)	422.63	8371.15	Dadia, Greece	Breeding, wintering, summering, on passage	Dadia FS, Year-round grazing livestock
					Studen Kladenets, Bulgaria	Breeding, wintering, summering, on passage	Studen Kladenets FS, Year-round grazing livestock
					Madjarovo, Bulgaria	Breeding, wintering, summering, on passage	Madjarovo FS, Year-round grazing livestock
					Kompsatos, Greece	Breeding, wintering, summering, on passage	Year-round grazing livestock
7	Western Greece, Greece	1H, 2H, 56, Alexis, C9, GD-22, GS, K3A-B2, K7A-B5, OX, (n=10)	363.54	7242.78	Akarnanika Mts/ Messolonghi/Embesos	Breeding, wintering,	Winter livestock grazing livestock
					Pindus Mts	Summering, on passage	Summer livestock grazing
