# Corrigendum: New insight into spatial ecology of Griffon Vulture (*Gyps fulvus*) on the Balkans provides opportunity for focusing conservation actions for a threatened social scavenger

**DOI:** 10.3897/BDJ.9.e73774

**Published:** 2021-09-01

**Authors:** Hristo Peshev, Atanas Grozdanov, Elena Kmetova–Biro, Ivelin Ivanov, Rigas Tsiakiris, Simeon Marin, Saša Marinković, Goran Sušić, Emanuel Lisichanets, Irena Hribšek, Zoran Karić, Sven Kapelj, Lachezar Bonchev, Emilian Stoynov

**Affiliations:** 1 Fund for Wild Flora and Fauna, 49 Ivan Mikhaylov Str., office 327, P.O.Box 78, www.fwff.org, pirin@fwff.org, Blagoevgrad, Bulgaria Fund for Wild Flora and Fauna, 49 Ivan Mikhaylov Str., office 327, P.O.Box 78, www.fwff.org, pirin@fwff.org Blagoevgrad Bulgaria; 2 South-West University „Neofit Rilski“, Faculty of Mathematics and Natural Sciences, Department of Geography, Ecology and Environmental Protection, Blagoevgrad, Bulgaria South-West University „Neofit Rilski“, Faculty of Mathematics and Natural Sciences, Department of Geography, Ecology and Environmental Protection Blagoevgrad Bulgaria; 3 Department of Zoology and anthropology, Faculty of Biology, Sofia University “St. Kliment Ohridski”, 8 Dragan Tsankov Blvd, zootribe@gmail.com, Sofia, Bulgaria Department of Zoology and anthropology, Faculty of Biology, Sofia University “St. Kliment Ohridski”, 8 Dragan Tsankov Blvd, zootribe@gmail.com Sofia Bulgaria; 4 Austrian Ornithological Central, Vienna, Austria Austrian Ornithological Central Vienna Austria; 5 Central European University, Department of Environmental Sciences and Policy, Vienna, Austria, Vienna, Austria Central European University, Department of Environmental Sciences and Policy, Vienna, Austria Vienna Austria; 6 Green Balkans – 9 Stara Planina Str., www.greenbalkans.org, officesz@greenbalkans.org, Stara Zagora, Bulgaria Green Balkans – 9 Stara Planina Str., www.greenbalkans.org, officesz@greenbalkans.org Stara Zagora Bulgaria; 7 Ministry of Environment and Energy, Forestry Service of Ioannina, Ioaninna, Greece Ministry of Environment and Energy, Forestry Service of Ioannina Ioaninna Greece; 8 Department of Ecology, Institute for Biological Research “Siniša Stanković” – National Institute of Republic of Serbia, University of Belgrade, Bulevar Despota Stefana 142, 11060, Belgrade, Serbia Department of Ecology, Institute for Biological Research “Siniša Stanković” – National Institute of Republic of Serbia, University of Belgrade, Bulevar Despota Stefana 142, 11060 Belgrade Serbia; 9 Ornithological Station Rijeka, Croatian Academy of Sciences and Arts, Rijeka, Croatia Ornithological Station Rijeka, Croatian Academy of Sciences and Arts Rijeka Croatia; 10 Nature Conservation Association - AQUILA, Kavadarci, Republic of North Macedonia Nature Conservation Association - AQUILA Kavadarci Republic of North Macedonia; 11 Natural History Museum of Belgrade, Njegoseva 51, Belgrade, Serbia Birds of Prey Protection Foundation, Bulevar despota Stefana 142, Belgrade, Serbia Natural History Museum of Belgrade, Njegoseva 51, Belgrade, Serbia Birds of Prey Protection Foundation, Bulevar despota Stefana 142 Belgrade Serbia; 12 Birds of Prey Protection Foundation, Bulevar despota Stefana 142, Belgrade, Serbia Birds of Prey Protection Foundation, Bulevar despota Stefana 142 Belgrade Serbia; 13 Association BIOM, Zagreb, Croatia Association BIOM Zagreb Croatia

## Corrigendum

This is a corrigendum of the article Peshev et al. (2021). Due to a software malfunction during the data processing, the values in Table 4, columns 4-5 are incorrect. The correct values are given here in Table 1.

The correct values must be taken in consideration also in the text, describing the results of Table 3 in the section "Defining vulture key zones in the Balkan Peninsula 1-7" and in the Abstract, where the sum of these values is included and is now corrected as follows: "The total home range 95% area of the Griffon Vulture population on the Balkans was estimated at 20,206.88 km² and the 50% core area at 851.73 km² (n = 48)."

In the section "Defining vulture key zones in the Balkan Peninsula" corrections were made in the numbers of tracked birds as follows: 3. Vrachanski Balkan Nature Park - corrected to "based on the location data of a total of 12 tracked birds.)"; 4. Eastern Balkan Mountain - corrected to "based on the location data of a total of 9 tracked birds)"; 6. Eastern Rhodopes - corrected to "(based on the location data of a total of 22 tracked birds)";

## Figures and Tables

**Table 1.** Correct values for Table 4, columns 4-5.

Vulture key zone/ Country	Area used by vutures, 50% core area, km^2^	Area used by vutures, 95% Home range, km^2^
Alpo - Adriatic, Austria/ Italy/ Croatia	144.45	3229.01
Western Serbia, Serbia	100.43	2356.42
Vrachanski Balkan Nature Park, Bulgaria	28.8	1197.21
Eastern Balkan Mountain, Bulgaria	13.76	626.3
Struma and Vardar Velleys, Bulgaria/ North Macedonia	107.3	3704.92
Eastern Rhodopes, Bulgaria/ Greece	236.54	4705.91
Western Greece, Greece	220.45	4387.11
